# Identifying and Quantifying Genotypes in Polyclonal Infections due to Single Species

**DOI:** 10.3201/eid1203.05057

**Published:** 2006-03

**Authors:** James M. Colborn, Ousmane A. Koita, Ousmane Cissé, Mamadou W. Bagayoko, Edward J. Guthrie, Donald J. Krogstad

**Affiliations:** * Tulane University Health Sciences Center, New Orleans, Louisiana, USA;; † University of Bamako, Bamako, Mali;; ‡ Agilent Technologies, Wilmington, Delaware, USA

**Keywords:** allotype, capillary electrophoresis, copy number, deletions, genotype, insertions, PCR

## Abstract

Simultaneous infection with multiple pathogens of the same species occurs with HIV, hepatitis C, Epstein-Barr virus, dengue, tuberculosis, and malaria. However, available methods do not distinguish among or quantify pathogen genotypes in individual patients; they also cannot test for novel insertions and deletions in genetically modified organisms. The strategy reported here accomplishes these goals with real-time polymerase chain reaction (PCR) and capillary electrophoresis. Real-time PCR with allotype-specific primers defines the allotypes (strains) present and the intensity of infection (copy number). Capillary electrophoresis defines the number of genotypes within each allotype and the intensity of infection by genotype. This strategy can be used to study the epidemiology of emerging infectious diseases with simultaneous infection by multiple genotypes, as demonstrated here with malaria. It also permits testing for insertions or deletions in genetically modified organisms that may be used for bioterrorism.

## Abstract 2

The combination of real-time PCR and capillary electrophoresis permits the rapid identification and quantification of pathogen genotypes.

Simultaneous infection with multiple pathogens of the same species occurs in human patients with HIV, hepatitis C, Epstein-Barr virus, dengue, tuberculosis, and malaria (*1**–**7*). However, available laboratory methods do not distinguish among pathogen genotypes in samples from individual patients. They do not permit the identification or quantitation of genotypes in samples with multiple pathogens of the same species, or the identification of size polymorphisms produced by insertions and deletions.

Conventional polymerase chain reaction (PCR) with agarose gel electrophoresis permits the identification of pathogen allotypes (strains) in human blood and tissue and an assessment of the sizes of their amplicons but does not define allotype copy number or genotype copy number. Real-time PCR permits identification and quantitation of allotypes (*8**,**9*) but does not permit the identification of genotypes within allotypes.

From the epidemiologic perspective, a molecular strategy to define the allotypes and genotypes of human pathogens and their copy numbers would permit one to study the dynamics of simultaneous infection with multiple genotypes in ways that have been impossible. For example, this knowledge could be used to identify novel genotypes (size polymorphisms) resulting from insertions and deletions at polymorphic loci.

From the bioterrorism perspective, a strategy to identify size polymorphisms (insertions and deletions) in critical regions of pathogen genomes would be of immense value. This information could be used to test for deletions in regulatory (suppressor) regions and for the insertion of new genes in regions controlled by strong promoters. Available methods do not permit the rapid identification of size polymorphisms within allotypes or the quantitation of individual pathogen genotypes.

To address this challenge, we used real-time PCR and capillary electrophoresis. Real-time PCR with allotype-specific primers permits one to define the allotypes present and their copy number (*8**,**9*). Capillary electrophoresis permits one to define individual genotypes within allotypes and genotype copy number. The combination of real-time PCR and capillary electrophoresis also permits the identification of insertions and deletions in potentially critical regions of pathogen genomes.

## Materials and Methods

### Collection of Patient Samples and Isolation of Pathogen DNA

Using fingersticks, filter paper blot samples (S & S #903 Blood Collection Cards, Schleicher & Scheuell Bioscience, Keene, NH, USA) containing 50 μL of blood were obtained from persons with *Plasmodium falciparum* infection in Mali. These samples were obtained in a prospective study of asymptomatic *P*. *falciparum* infection in the village of Missira (160 km northwest of Bamako), after review and approval by the University of Bamako Institutional Review Board (IRB) in Bamako and the Tulane University IRB in New Orleans. Before obtaining informed consent from the participants (after IRB reviews and approvals), the protocol was reviewed with the chief and elders of the village and the women's council. After those additional reviews and approvals, informed consent was obtained from persons >18 years of age and from the parents and guardians of children <17 years of age before obtaining blood samples. DNA was isolated from filter paper blots and blood samples by using the QIAamp DNA Mini Kit (Qiagen, Valencia, CA, USA).

Control template DNA was obtained from cultured parasites (*10*) by using the QIAamp DNA Blood Mini Kit (Qiagen). Cloned isolates used as controls for the 4 known allotypes of the polymorphic block 2 region of merozoite surface protein 1 (*msp1*) were Indochina I/CDC for MAD20, Haiti 135 for K1, 7G8 for RO33, and OK/JC 5 for MAD20/RO33 hybrid allotype parasites (*11**–**13*). DNA template concentrations were estimated from standard curves by plotting the fluorescence of 5 DNA standards with concentrations of 1 μg/mL (1,000 ng/ml), 100 ng/mL, 10 ng/mL, 1 ng/mL, and 0 ng/mL (blank or negative control) vs. the log_10_ of template DNA concentration by using PicoGreen dye (Molecular Probes, Eugene, OR, USA) with the VersaFluor fluorometer (Bio-Rad, Hercules, CA, USA).

### Primer and Probe Design for Real-time PCR

Primers and probes were designed by using the Beacon Designer software, version 2.03, Premier (Biosoft International, Palo Alto, CA, USA) (available from http://www.premierbiosoft.com/molecular_beacons/), in combination with manual manipulation. The primers and probes used to amplify the K1, MAD20, RO33, and hybrid (MAD20/RO33) allotypes of the block 2 region of *msp1* and the internal control gene (erythrocyte-binding antigen 175, *eba175*) in *P*. *falciparum* are listed in Table 1, together with information on fluorophores, melting temperatures, final reactant concentrations, and the observed ranges of amplicon sizes. Unlabeled primers and fluorophore-labeled probes were obtained from Integrated DNA Technologies (Coralville, IA, USA), LUX-labeled primers from Invitrogen (Carlsbad, CA, USA); and the Cy5-labeled probe for *eba175* from Biosearch Technologies (Novarto, CA, USA).

**Table 1:** Primers and probes used for real-time polymerase chain reaction*

Primer/probe sequence		Sizes (bp)	T_m_ (°C)	Fluorophore, quencher	Final reactant concentration (nmol/L)
Parasites with K1 and hybrid sequences in block 2 of *msp1*					
	K1F 5´-AGGTGCAAGTGCTCAAAGTG-3´	108–171	50.3	Texas Red, BHQ-2	100
	K1R 5´-CACCAGATGAAGTATTTGAACG-3´		49.2		100
	PROBE: 5´-AAGTGGTACAAGTCCATCATCTCGT-3´		54.9		300
	Hybrid F 5´-GAAGGAACAAGTGGAACAGC-3´	96–168	48.7	Cy5, BHQ-2	200
	Hybrid R 5´-GCAGCACCTGGAGATCTTATA-3´		48.8		200
	PROBE: 5´-TTCACTTATTTCCCCATGAGCCCC-3´		54.2		400
Primers for positive control (EBA175)					
	EBA175 F 5´-GGTTATTCAACTAAGGCAGAA-3´	95	46.0	Cy 5, BHQ-3	100
	EBA175 R 5´-TCCACCATTCTTTTCTAAAATTTT-3´		50.6		100
	PROBE: 5´-TCATTTCCCATAGCAAGATGTCC		60.0		100
LUX primers (MAD 20, RO33)					
	MAD20 F 5´-AATGAAGGAACAAGTGGAAC-3´	52–205	48.7	FAM	200
	MAD20 R 5´-GAATTATCTGAAGGATTTGTACG-3´		47.4	(reverse)	200
	RO33 F 5´-GCAGATGCTGTAAGTACTCAA-3´	148	44.8	JOE	200
	RO33 R 5´-GCAGCACCTGGAGATCTTATA-3´		44.8	(forward)	200

*msp1, merozoite surface protein 1, EBA175, erythrocyte-binding antigen 175; T_m_, melting temperature.

### Real-time PCR Amplification of Pathogen DNA

Real-time PCR was performed with the iCycler (Bio-Rad) by using the amplification conditions described below (Table 1) with a 2× multiplex-specific master mix (Qiagen) and 3-μL aliquots of template DNA. Reaction mixtures were supplemented with 2.5 U recombinant Taq polymerase (Invitrogen) and subjected to an initial denaturation at 95°C for 15 min, followed by 45 cycles of denaturation at 94° C for 30 s, annealing at 53°C for 90 s, and extension at 72°C for 90 s. Fluorescence measurements were obtained during the annealing step with TaqMan probes (K1 and MAD20/RO33 hybrid allotypes, and the *eba175* internal control), and during the elongation step with LUX primers (MAD20 and RO33 allotypes). Each sample was tested in quadruplicate. Two samples were used to define the allotypes present and their copy number with the iCycler; the other 2 samples were removed from the iCycler during the exponential (logarithmic) stage of amplification for capillary electrophoresis to define the genotypes present and genotype copy number.

### Template Specificity and Optimization of Multiplex PCR

Template specificity was tested for each primer probe set with the 4 control template DNAs (from Indochina I, Haiti 135, 7G8 and hybrid MAD20/RO33 parasites). Amplicons of the expected sizes were obtained with matched template and primer probe sets; no amplicons were obtained with unmatched template and primer probe sets. Negative controls likewise yielded no amplicons. DNA extracted from specimens without parasites was used to control for primer-primer and primer-probe interactions, and other potential causes of false-positive PCR results. After establishing specificity, the reaction conditions were optimized by defining the efficiencies of each primer probe set using a series of 10-fold dilutions with each control template DNA. These efficiencies were then matched to the efficiencies obtained with the multiplex PCR to adjust the final primer and probe concentrations so the efficiencies of the multiplex PCR matched those of the individual PCRs.

### PCR Amplification and Allotype Quantitation

Standard curves were generated by using 10-fold dilutions of template DNA (3-μL aliquots) from each of the control parasites to estimate the initial copy numbers of the 4 allotypes in each sample. The standard curves (regression lines) for each allotype, the resulting reaction efficiencies, threshold cycle (C_T_) values and estimates of initial copy numbers were calculated by using the iCycler Software (Bio-Rad).

### Capillary Electrophoresis and Genotype Quantitation

To estimate amplicon size (base pairs) and copy number for each genotype, 2 replicates were removed from the iCycler for each sample during the logarithmic amplification stage, as determined by real-time relative fluorescence unit (RFU) data, and stopped with 0.5 mol/L EDTA. For each reaction, two 1-μL aliquots of the real-time PCR reaction mixture were loaded onto a DNA 500 Lab Chip (Agilent Technologies, Waldbronn, Germany) and run on the Bioanalyzer 2100 (Agilent Technologies), according to the manufacturer's instructions. With capillary electrophoresis using the DNA 500 Lab Chip, a linear relationship was shown between amplicon size and elution time (*r^2^*>0.998, p<0.001 for amplicons from 25 bp to 400 bp; data not shown). The copy numbers for each genotype were calculated from the molarities provided by the Agilent software. These calculations are based on the observation that the concentration of each amplicon is proportional to its peak area on the electropherogram.

## Results

### Real-time PCR To Identify Pathogen Allotypes

**Figure 1:**
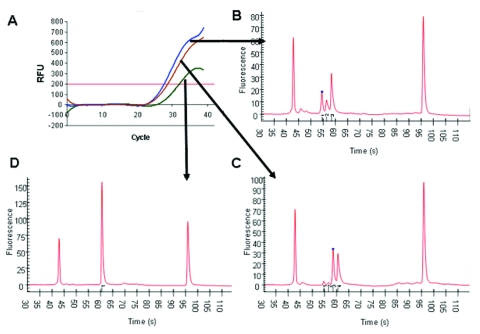
Use of capillary electrophoresis to identify multiple genotypes within single allotypes amplified by real-time polymerase chain reaction. Panel A shows the relative fluorescence values for 3 samples from infected patients by using primers specific for the K1 allotype of merozoite surface protein 1 (*msp1*). Panels B, C, and D show that those samples contained 3, 4, and 1 different K1 genotype parasites, respectively, identified by amplicons of 106, 124, and 142 bp (panel B), 105, 124, 142, and 160 bp (panel C), and 160 bp (panel D), respectively. The first and last peaks on each electropherogram are the 15- and 600-bp standard markers used to define the sizes of the unknown amplicons.

Real-time PCR with allotype-specific primers permits the amplification of individual allotypes in specimens from infected human subjects (first 3 columns of Table 2, and Figure 1, panel A). Based on control specimens containing only 1 allotype and on negative controls, this strategy is specific. Based on filter paper blots for specimens containing >100 parasites/μL, it is sensitive. However, real-time PCR with allotype-specific primers does not distinguish among (identify) genotypes within allotypes (Figure 1). This is because real-time PCR cannot identify size polymorphisms, whether they result from natural events such as the spontaneous addition and deletion of tripeptide repeats in malaria parasites (*14*) or deliberately malevolent manipulation of microorganisms in the laboratory as potential agents of bioterrorism.

**Table 2:** Distribution of allotypes and genotypes in 1 blood sample*

Allotype	Amplicon size, bp (no. repeats)	Allotype copy no.	Proportions of individual genotypes (%)	Genotype copy no.
MAD20	184 (15)	1.38 × 10^3^	100	1.38 × 10^4^
K1	106 (9)	1.53 × 10^4^	19	2.90 × 10^3^
K1	124 (11)		61	9.30 × 10^3^
K1	133 (12)		7	1.10 × 10^3^
K1	151 (14)		13	1.99 × 10^3^
Hybrid	159 (7)	4.67 × 10^5^	100	4.67 × 10^5^
RO33	NA	NA	0	0

*NA, not available.

### Optimization of Real-time PCR

Estimates of efficiency (the degree to which replication increases the number of amplicons by the expected 2-fold increment [100% efficiency] in each cycle) indicate that the efficiency of the real-time PCR assays performed in these studies was excellent (90%–100%). In addition, efficiencies of reactions in multiplex did not differ significantly from individual reaction efficiencies, or from other reaction efficiencies in multiplex.

### Reproducibility of Copy Number (Threshold Cycle, C_T_) Estimates

Data for estimates of copy number were based on the amplification of block 2 of *msp1* from *P*. *falciparum* malaria parasites (Table 2). The reproducibility of C_T_ estimates was examined separately for exemplary 93- and 154-bp amplicons, and found to have means of 27.99 and 28.62 cycles, respectively, with standard deviations of 0.34 and 0.13 cycles (i.e., coefficients of variation [CVs] of 1.2% and 0.5% for these 2 amplicons, n = 10 for each).

### Capillary Electrophoresis To Identify Pathogen Genotypes

**Figure 2:**
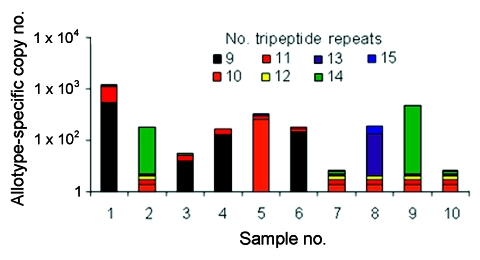
Copy numbers for genotypes of the K1 allotype in 10 field samples. Distribution of K1 genotypes within the 8 patients whose samples yielded amplicons with K1-specific primers (Table 4). These results indicate that most infected persons had >2 allotypes. In addition, persons with K1 allotype parasites had a high degree of genotypic complexity, that is, capillary electrophoresis showed up to 4 distinct K1 genotypes in the blood of individual patients at the same time.

**Figure 3:**
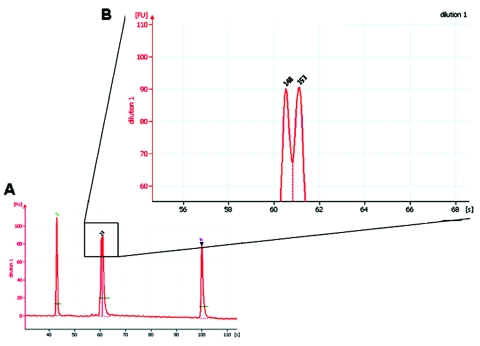
Capillary electrophoresis separation of polymerase chain reaction amplicons differing by 5 bp.

In contrast to real-time PCR, which identifies only allotypes, capillary electrophoresis identifies genotypes within allotypes (based on size polymorphisms) in samples from persons (Figure 1, panels B–D). Across participants (in groups of samples), this method permits one to identify the spectrum (range) of genotypes in the population (data for samples from 10 persons infected with *P*. *falciparum* are presented as an example in Figure 2 and Table 3). The reproducibility of capillary electrophoresis is sufficient to separate amplicons that differ by >5 bp. This conclusion was based on a comparison of amplicons containing 148 and 153 bp with elution times on electropherograms of 60.51 and 61.11 s, respectively (Figure 3).

**Table 3:** Copy number for amplification of *msp1* allotypes in field samples*

	Allotype			
Field sample	MAD20	RO33	K1	Hybrid
1	1,380	0	15,300	467,000
2	0	361,000	342	0
3	813	0	25.53	0
4	138.4	0	261.4	0
5	25.65	0	1,090	101
6	6.71	0	323.7	29.7
7	143	0	22.6	75.7
8	548	0	361	0
9	717	0	2,320	11.9
10	2,330	226	44.6	178

*Distribution of allotype-specific copy numbers for samples from 10 subjects infected with *Plasmodium falciparum*. Both MAD20 and K1 allotype parasites were present in 8 of the 10 subjects, MAD/RO hybrid allotypes were present in 4, and RO33 was present in 1.

### Reproducibility of Genotype Copy Number Estimates

Based on the electropherograms, the reproducibility of peak area measurements and estimates of genotype copy number was excellent. CVs varied from 0.13% to 0.45% for amplicon concentrations between 10 nmol/L and 80 nmol/L (n = 12 replicates at each of 4 template concentrations of a 95-bp amplicon from 10 nmol/L to 80 nmol/L, data not shown).

### Real-time Fluorescence in Relation to Peak Area

**Figure 4:**
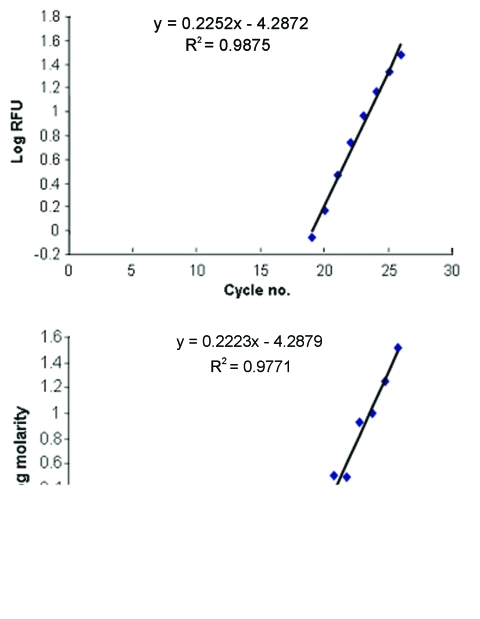
Comparison of amplicon concentration based on relative fluorescence from real-time polymerase chain reaction with peak area from capillary electrophoresis.

The slopes of increasing fluorescence based on real time PCR with the iCycler were indistinguishable from the increasing peak areas on the electropherogram (Figure 4, panels A and B, slopes of 0.2252 and 0.2223, p > 0.5). The similarity of these slopes (based on different parameters) indicates that increases in RFUs are directly proportional to increases in amplicon concentration (molarity). This result permits one to extrapolate from allotype copy number to genotype copy number based on peak area.

### Field Samples from Persons with Polyclonal Infections

Three of the 4 known *P*. *falciparum* allotypes have size polymorphisms within block 2 of *msp1*. K1, MAD20, and hybrid MAD20/RO33 allotype parasites have size polymorphisms because they contain tripeptide repeats within block 2 of *msp1*; RO33 does not have size polymorphisms because it does not have tripeptide repeats (*15*). These size polymorphisms are evident for K1 in a sample from a single person (Table 2) and for K1, MAD20, and hybrid MAD20/RO33 parasites in samples from 10 persons (Figure 2 and Table 3).

## Discussion

### Simultaneous Infection and Detection of Genetically Modified Organisms

Studies by a number of investigators have shown that simultaneous infection with multiple pathogens (genotypes) of the same species occurs in patients with HIV, hepatitis C, Epstein-Barr virus, dengue, tuberculosis, and malaria (*1**–**7*) and have identified deletions and insertions (genotypes) due to tandem repeats in cytomegalovirus (*15*). Because pathogen genotypes based on insertions and deletions are common, the strategy reported here is potentially applicable to all microbial human pathogens. This complexity of infection is likely to be important in the pathogenesis and transmission of many emerging infectious diseases. For example, epidemiologically and clinically meaningful events such as severe disease and antimicrobial drug resistance are likely to be driven by competition among pathogen genotypes in vivo (by the virulence and antimicrobial susceptibility/resistance determinants of the predominant genotypes) and may also affect transmission.

In addition to block 2 of *msp1* in *P*. *falciparum*, other examples of natural sequence variation detectable by using real-time PCR and capillary electrophoresis (variations >5 nucleotides/bp) include duplications and deletions in the 3´ noncoding regions (NCRs) of dengue (*16*) and yellow fever (*17*) and insertions and deletions in the *env* gene of HIV (*18**,**19*). For *Mycobacterium tuberculosis*, examples include variation in the tandem repeats within *IS*6110 (*20*), variable numbers of tandem repeats (VNTRs) (*21*), and genomic deletions (*22*). For select agents, examples include variation in VNTRs (multiple locus VNTR analysis) in *Bacillus anthracis* (*23**,**24*), similar differences in *Yersinia pestis* (*25*), and insertions, deletions, and variation in the inverted terminal repeat region and the coding region of the smallpox virus (*26**,**27*) (Table 4).

**Table 4:** Evidence for insertions, deletions, and repeats in human pathogens*

Pathogen	Genomic site of variation	Observed size variations	Reference
*Plasmodium falciparum*	Block 2 variable region of merozoite surface protein 1 (*msp1*), PCR	150–200 bp with multiple 9-bp insertions and deletions based on number of tripeptide repeats	(*14*)
Dengue	3´ NCR after the NS5 stop codon	2–14 and 75-nt deletions, 4 copies of 8-nt imperfect repeat	(*16*)
Yellow fever virus	3´ NCR	216-nt duplication, 40-nt deletion (repeat hairpin motif)	(*17*)
HIV	*env* gene	35- and 48-nt insertions, 21- and 36-nt deletions	(*18*)
	*gp120* V3 and V4 loops	9- and 12-nt deletions	(*19*)
*Mycobacterium tuberculosis*	Novel *IS*6110 insertions†	36-bp DRs interspersed with variable spacers for DVRs	(*20*)
	VNTRs	Repeating units of 53–79 bp with 16–17 copies	(*21*)
	Genomic deletions	Based on genomic microarrays	(*21*)
*Bacillus anthracis*	MLVA†	Variations of 12, 9, 18, 72, and 5 bp for MLVA markers *vrrA*, *vrrB1*, *vrrB2*, *vrrC2*, and CG3	(*23*)
	Subtyping of 2001 bioterrorism organism	All isolates were genotype 62	(*24*)
*Yersinia pestis*	MLVA† with 25 markers for tandem repeat loci with 9–60 bp repeats of 3–36 units	Amplicon sizes for complete alleles ranging from 119 to 786 bp	(*25*)
Smallpox virus	Coding regions of the viral genome	Variable numbers of 9- and 21-bp repeats (n = 5–31 and 15–38, respectively), insertions of 32 and 464 bp and a 251-bp deletion	(*26*)
	Inverted terminal repeats between nonrepetitive elements 1 and 2 (NR1, NR2)	0–4 copies of a 69-bp sequence	
	Potential virulence proteins	Smallpox inhibitor of complement enzymes, chemokine-binding protein II, and Z-DNA binding protein	(*27*)

*PCR, polymerase chain reaction; NCR, noncoding region; DRs, direct repeats; DVRs, direct variant repeats; VNTRs, variable numbers tandem repeats; MLVA, multiple locus VNTR analysis; msp1, merozoite surface protein 1. †Exists at multiple sites within the pathogen genome.

In addition, disease-producing agents may be modified in the laboratory to increase their virulence or to introduce antimicrobial drug resistance for bioterrorist events (*28**–**30*). However, available methods are inadequate to rapidly diagnose and quantitate simultaneous infection with multiple pathogens (genotypes) of the same species or identify insertions and deletions in critical regions of pathogen genomes. The results reported here provide a strategy to address these issues based on real-time PCR and capillary electrophoresis.

### Real-time PCR To Identify and Quantitate Pathogen Allotypes

As demonstrated here and elsewhere, allotype-specific primers permit one to identify the pathogen allotypes in a specimen (*1**–**7*). In addition, real-time PCR may be used to quantitate the numbers of microorganisms in a specimen. Because the relationship between the number of cycles necessary to reach the C_T_ and the log_10_ of copy number is linear, real-time PCR can be used to estimate the initial amount of template DNA (copy number) (*8**,**9*).

### Capillary Electrophoresis To Identify and Quantitate Pathogen Genotypes

In contrast to real-time PCR (in which all amplicons [genotypes] are examined together in the same well once each cycle), capillary electrophoresis detects the amplicons from each genotype as they pass a fluorescence or absorbance detector. This is accomplished by separating dsDNA amplicons based on their size (base pairs) by using a charged electrical field to drive the dsDNA polyanions to the detector at the anode. Because this separation is driven by the ratio of the electrical driving force to the mass of each amplicon, the rate of movement to the anode is inversely proportional to mass (size in base pairs). Thus, smaller amplicons travel faster and have shorter retention times on the electropherogram (Figure 1, panels B–D).

### Detection of Artificial-size Polymorphisms

The results reported here demonstrate that capillary electrophoresis is sufficiently sensitive to detect insertions and deletions >5 bp in size. This finding means that capillary electrophoresis is more than sufficiently sensitive to detect biologically significant insertions and deletions in genetically modified organisms (*23**–**30*). Thus, it provides an open-ended strategy to test for genetically modified organisms, by testing for size polymorphisms at critically important sites in the pathogen genome, e.g., at sites related to pathogenicity (virulence) or antimicrobial resistance.

### Advantages, Limitations, and Potential Pitfalls

The advantages of real-time PCR followed by capillary electrophoresis are that it can be performed without waiting days or weeks for cultures to grow and that it detects pathogens that do not grow in conventional culture media or under standard conditions (*31*). In addition, as noted above (*14*), sequence information is enormously helpful in selecting loci within the genome likely to have insertions and deletions and interpreting the results obtained. Although insertions, deletions, and single nucleotide polymorphisms (SNPs) produce detectable changes in melting curves (*32**,**33*), melting curves are qualitative rather than quantitative. In addition, melting curves alone cannot identify specific insertions, deletions, or quasispecies (SNPs) without the addition of probes for the affected target region of the genome or the use of PCR (*34**,**35*). Finally, because the strategy reported here tests for size polymorphisms, it does not require prior knowledge of the specific sequences that may have been introduced into (or deleted from) the pathogen genome to identify genetically modified organisms. However, this strategy does have 3 limitations.

First, sequences identical to (or cross-reactive with) host sequences cannot be used as targets because blood and tissue specimens are inevitably contaminated with host DNA (this issue can be resolved by searching the GenBank database). Second, the threshold of detection for genetically modified organisms is the addition (or removal) of sequences >5 bp (based on the sensitivity of capillary electrophoresis), i.e., point mutations (SNPs = quasispecies) (*36**–**38*) cannot be detected with this strategy. As a result, this method is likely to be of greater value for organisms with dsDNA genomes such as bacteria, eukaryotic parasites, and dsDNA viruses (in which quasispecies are less common because of more accurate replication) than for organisms with single negative-stranded RNA genomes (in which quasispecies are more common because their replication depends on the error-prone reverse transcriptase—HIV, hepatitis C, hepatitis B) (*39**,**40*). Third, capillary electrophoresis may need to be performed separately for each allotype to avoid confusion between amplicons of similar size from different allotypes (Figures 1,2, and 3).

## Conclusions

The strategy reported here can be used for epidemiologic studies of simultaneous infection with multiple pathogens (genotypes) of the same species in emerging infectious diseases and for the rapid identification of select agents that have been genetically modified to increase their virulence or antimicrobial drug resistance.
